# Multidimensional cardiac functional phenotyping beyond ejection fraction: a multimodality imaging framework for cardiovascular care

**DOI:** 10.3389/fcvm.2026.1868144

**Published:** 2026-07-07

**Authors:** Yuan Li, Yujian Liu

**Affiliations:** 1 Department of Ultrasound, Zigong Fourth People’s Hospital, Zigong, Sichuan, China; 2 Department of Radiology, Zigong First People’s Hospital, Zigong, Sichuan, China

**Keywords:** artificial intelligence, cardiac function, diastolic function, functional phenotyping, multimodality imaging, myocardial deformation, tissue characterization

## Abstract

Cardiac functional assessment has traditionally relied on left ventricular ejection fraction (LVEF), but this single volumetric index cannot fully capture the complexity of cardiac physiology. Contemporary multimodality imaging enables cardiac function to be evaluated across five interrelated domains: pump function, myocardial deformation, diastolic function, flow dynamics, and tissue characterization. Integrating biomarkers across these domains may provide a more pathophysiologically grounded interpretation of cardiac dysfunction than LVEF alone. Clinically, multidimensional functional phenotyping may refine disease characterization and risk stratification in conditions such as heart failure with preserved ejection fraction, cardiomyopathies, and valvular heart disease. This review proposes a five-dimensional, phenotype-oriented multimodality imaging framework for cardiovascular care and emphasizes a selective stepwise escalation strategy from standard echocardiography to advanced echocardiographic analysis, cardiac magnetic resonance, computed tomography, nuclear imaging, and selected flow-based assessment, particularly 4D flow cardiovascular magnetic resonance, when clinically indicated. The framework is intended to support clinically actionable interpretation rather than indiscriminate use of multiple imaging modalities. We also discuss practical barriers to implementation, including cost-effectiveness, workflow burden, vendor variability, limited standardization of 4D flow cardiovascular magnetic resonance, and the need for interpretable and externally validated artificial intelligence-based integration.

## 1 Introduction

Cardiac functional assessment is central to the diagnosis and management of cardiovascular disease. For decades, LVEF has served as the cornerstone of systolic evaluation owing to its accessibility, reproducibility, and prognostic value across multiple imaging modalities, and it remains integral to current clinical guidelines (1–3).

However, the LVEF-centered paradigm has important limitations. LVEF is relatively insensitive to early or subclinical myocardial dysfunction and may remain normal in heart failure with preserved ejection fraction (HFpEF) and cardiotoxicity (2, 4, 5). It also underestimates contributions from the right ventricle and left atrium to overall cardiac performance (6, 7) and provides no information on myocardial tissue composition or intracardiac flow dynamics (8, 9).

Against this background, cardiac functional assessment is shifting from a single-parameter approach toward multidimensional integrative phenotyping (3, 4, 10). Although the complementary roles of echocardiography, cardiac magnetic resonance (CMR), computed tomography (CT), and nuclear imaging are increasingly recognized, their results are typically reported in modality-specific silos without a unifying cross-dimensional interpretive structure. In current practice, multimodality imaging is often applied in an *ad hoc* manner: additional modalities are selected to answer individual clinical questions, but the process is not always guided by a clear hierarchy of escalation from first-line echocardiography to advanced imaging. As a result, clinicians may lack a structured approach for determining when escalation is warranted, how abnormalities across functional dimensions should be integrated, and how multiparametric outputs should be translated into clinically actionable phenotypes. What is needed is not simply more measurements, but a practical structure for deciding which measurements matter in a given clinical context.

This review synthesizes multimodality imaging evidence into a five-dimensional framework encompassing pump function, myocardial deformation, diastolic function, flow dynamics, and tissue characterization, further refined by multi-chamber integration. Relevant literature was identified from PubMed/MEDLINE, prioritizing clinical guidelines, consensus statements, systematic reviews, and high-impact original research published from 2015 onward; seminal earlier studies were included where foundational. A structured summary of the evidence base supporting each functional dimension is provided in Table 1. Unlike modality-specific guidelines or isolated parameter reviews, this framework organizes imaging assessment around functional phenotypes and proposes a selective, stepwise escalation strategy in which standard echocardiography serves as the first-line gatekeeper. Advanced echocardiography, CMR, CT, nuclear imaging, or flow-based assessment, particularly 4D flow cardiovascular magnetic resonance (4D flow CMR), should be added only when the result is expected to clarify diagnosis, refine risk stratification, or influence management. The goal is to make multimodality imaging more selective and interpretable, not more indiscriminate. Practical barriers to implementation, including cost-effectiveness, vendor variability, workflow integration, and the interpretability requirements of artificial intelligence (AI)-based approaches, are addressed explicitly.

**Table 1:** Evidence base and representative reference anchors for the five-dimensional cardiac functional phenotyping framework.

Functional dimension	Representative biomarkers	Main modalities	Evidence base	Clinical role	Reference anchors and key limitations
Pump function	LVEF, RVEF, stroke volume, cardiac output, ventricular volumes	Echocardiography, CMR, cardiac CT	HF guidelines; chamber quantification recommendations; right-heart imaging consensus (1–4, 6, 12, 19, 20)	Disease classification, treatment eligibility, baseline risk stratification, therapy monitoring	LVEF <50% often indicates systolic dysfunction; TAPSE <17 mm and FAC <35% support RV systolic dysfunction. Load dependence and intermodality variability remain major limitations.
Myocardial deformation	GLS, GCS, GRS, RV free-wall strain, LA strain	STE, CMR-FT, CT-FT	Strain consensus statements; cardio-oncology guidelines; cohort and meta-analytic evidence (5, 14, 21–31)	Subclinical dysfunction detection, cardio-oncology surveillance, chamber-specific risk assessment	Normal LV GLS magnitude is commonly around −18% to −22%; >15% relative reduction in GLS magnitude supports CTRCD. Vendor/software dependence remains important.
Diastolic function	E/A, e′, E/e′, LAVI, TR velocity, LA reservoir strain	Echocardiography, advanced echocardiography, adjunctive CMR	Diastolic function guidelines; HFpEF and LA strain studies (1–3, 7, 10, 15, 18, 23, 30, 32–35)	Filling-pressure estimation, HFpEF characterization, atrioventricular coupling assessment	E/e′ > 14 and LAVI >34 mL/m^2^ support elevated filling pressure. Interpretation is affected by age, rhythm, loading conditions, mitral disease, and image quality.
Flow dynamics	Forward flow, regurgitant volume, shunt fraction, vortex pattern, flow stasis, TKE	PC-CMR, 4D flow CMR, Doppler echocardiography	4D flow CMR consensus; valvular/congenital disease studies; intracardiac flow reviews (9, 16, 36–39)	Flow burden quantification, regurgitation/shunt assessment, complex valvular or congenital disease evaluation	PC-CMR is established for flow quantification; 4D flow CMR lacks universal diagnostic thresholds and remains limited by acquisition time, post-processing, standardization, and reimbursement.
Tissue characterization	LGE, native T1/T2 mapping, ECV, edema, fibrosis, infiltration, PET uptake	CMR, nuclear imaging/PET, adjunctive cardiac CT	CMR tissue characterization reviews; LGE/ECV prognostic studies; amyloidosis, myocarditis, and cardiomyopathy studies (8, 17, 20, 40–51)	Myocardial substrate diagnosis, scar/fibrosis/inflammation detection, risk stratification	LGE is abnormal in the appropriate clinical context; ECV >30% is often considered elevated but is disease-, scanner-, and protocol-dependent. Availability and contrast/PET constraints limit general use.

Reference anchors are provided to improve clinical interpretability and should not be interpreted as universal diagnostic thresholds. Values vary according to disease context, imaging modality, vendor platform, acquisition protocol, post-processing software, loading conditions, and patient characteristics.

4D flow CMR, four-dimensional flow cardiac magnetic resonance; CMR, cardiac magnetic resonance; CMR-FT, cardiac magnetic resonance feature tracking; CT, computed tomography; CT-FT, computed tomography feature tracking; CTRCD, cancer therapy-related cardiac dysfunction; ECV, extracellular volume; FAC, fractional area change; GCS, global circumferential strain; GLS, global longitudinal strain; GRS, global radial strain; HF, heart failure; LA, left atrial; LAVI, left atrial volume index; LGE, late gadolinium enhancement; LV, left ventricular; LVEF, left ventricular ejection fraction; PC-CMR, phase-contrast cardiac magnetic resonance; PET, positron emission tomography; RV, right ventricular; RVEF, right ventricular ejection fraction; STE, speckle-tracking echocardiography; TAPSE, tricuspid annular plane systolic excursion; TKE, turbulent kinetic energy; TR, tricuspid regurgitation.

## 2 Conceptual evolution of cardiac functional assessment: from ejection fraction to functional phenotyping

### 2.1 Conventional paradigm: ejection fraction-centered assessment

LVEF, derived from end-diastolic and end-systolic volumes, has been widely applied across echocardiography, CMR, and CT, serving as a cornerstone for disease classification, therapeutic decision-making, and prognostic assessment (1–4). However, as a global and integrative parameter, LVEF does not directly capture regional myocardial mechanics, diastolic properties, intracardiac flow patterns, or tissue characteristics (8, 9, 11), and may therefore obscure functional abnormalities—particularly in early disease stages or conditions with heterogeneous myocardial involvement (4, 5, 10).

### 2.2 A five-dimensional integrative framework for functional phenotyping

Advances in cardiovascular imaging have shifted the understanding of cardiac function from a single global metric to a multidimensional construct (3, 12, 13). Based on these developments, cardiac function can be conceptualized across five interrelated domains. Pump function encompasses global ventricular output metrics, including LVEF, stroke volume, and cardiac output (1, 4). Myocardial deformation characterizes intrinsic myocardial mechanics through strain and strain rate measurements (1, 6, 14). Diastolic function reflects ventricular filling properties and loading conditions (1, 7, 15). Flow dynamics describes intracardiac flow patterns and energetic efficiency (9, 16). Tissue characterization assesses myocardial structure and composition, including fibrosis, edema, and infiltration (8, 17). The key imaging biomarkers, representative modalities, evidence base, clinical value, and major limitations for each dimension are summarized in Table 1.

These domains constitute an interconnected system rather than independent parameters; their combination defines disease-specific functional phenotypes. Patients with similar ejection fractions may therefore exhibit markedly different functional profiles (3, 4, 10). Importantly, the framework does not imply that every patient requires assessment across all dimensions or modalities. Its role is to guide selective use and interpretation of imaging information according to the clinical question.

### 2.3 Clinical implications and transition toward multidimensional assessment

**Figure 1:**
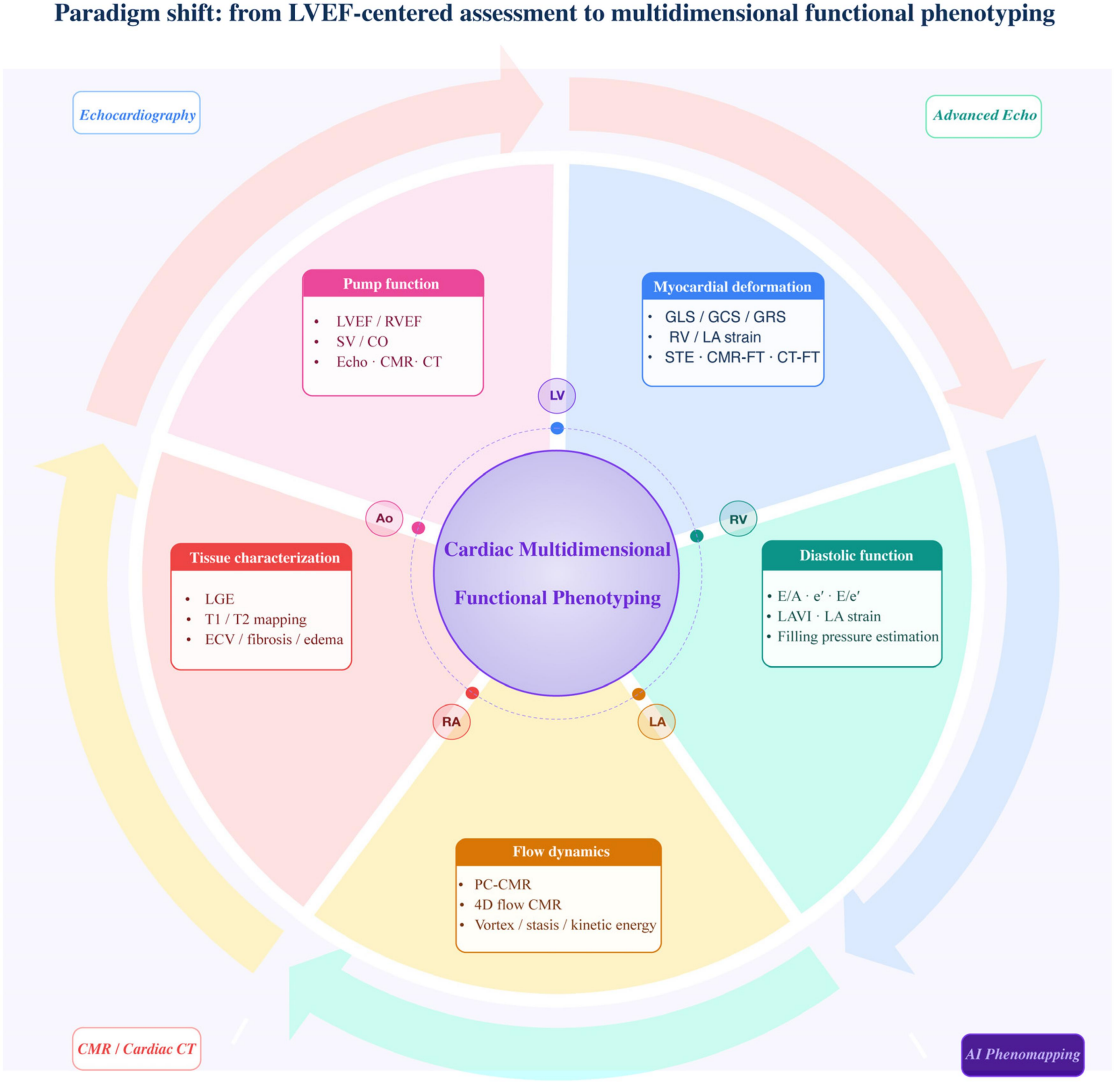
Five-dimensional framework for cardiac multidimensional functional phenotyping.

Functional phenotyping addresses clinical scenarios inadequately captured by traditional metrics, such as preserved ejection fraction with early myocardial deformation abnormalities, or impaired diastolic filling in the absence of overt systolic decline (4, 10, 18). It also supports multimodality imaging integration, enabling complementary insights across functional domains and providing the foundation for the detailed examination of each dimension in subsequent sections (Figure 1).

The framework depicts the conceptual shift from conventional LVEF-centered assessment to multidimensional functional phenotyping. Five interrelated domains—pump function, myocardial deformation, diastolic function, flow dynamics, and tissue characterization—are organized within a unified functional network, further refined by multi-chamber integration involving the left ventricle (LV), right ventricle (RV), left atrium (LA), right atrium (RA), and great-vessel interaction. Echocardiography denotes first-line conventional assessment, whereas advanced echocardiography (Advanced Echo) refers to strain imaging, 3D echocardiography, and advanced chamber-function analysis. CMR is positioned adjacent to the tissue characterization domain to reflect its central role in late gadolinium enhancement, parametric mapping, and extracellular volume assessment, whereas cardiac CT provides complementary anatomical and selected functional information. AI-enabled phenomapping indicates cross-domain integration of quantitative imaging biomarkers when supported by validated, interpretable, and clinically actionable models.

## 3 Multidimensional assessment of cardiac function based on multimodality imaging

### 3.1 Pump function

Pump function reflects the heart's ability to generate forward blood flow, quantified primarily by LVEF, right ventricular ejection fraction (RVEF), stroke volume, and cardiac output (1–4). Echocardiography—particularly two-dimensional (2D) and three-dimensional (3D) techniques—remains the first-line modality due to its wide availability and real-time capability; the biplane Simpson method is standard for LVEF, while 3D echocardiography improves volumetric accuracy (3, 19). CMR is the reference standard for ventricular volume quantification, especially for the right ventricle and complex geometries (6, 17), and electrocardiographically gated CT can provide reliable complementary volumetric data in selected clinical scenarios (20).

**Figure 2:**
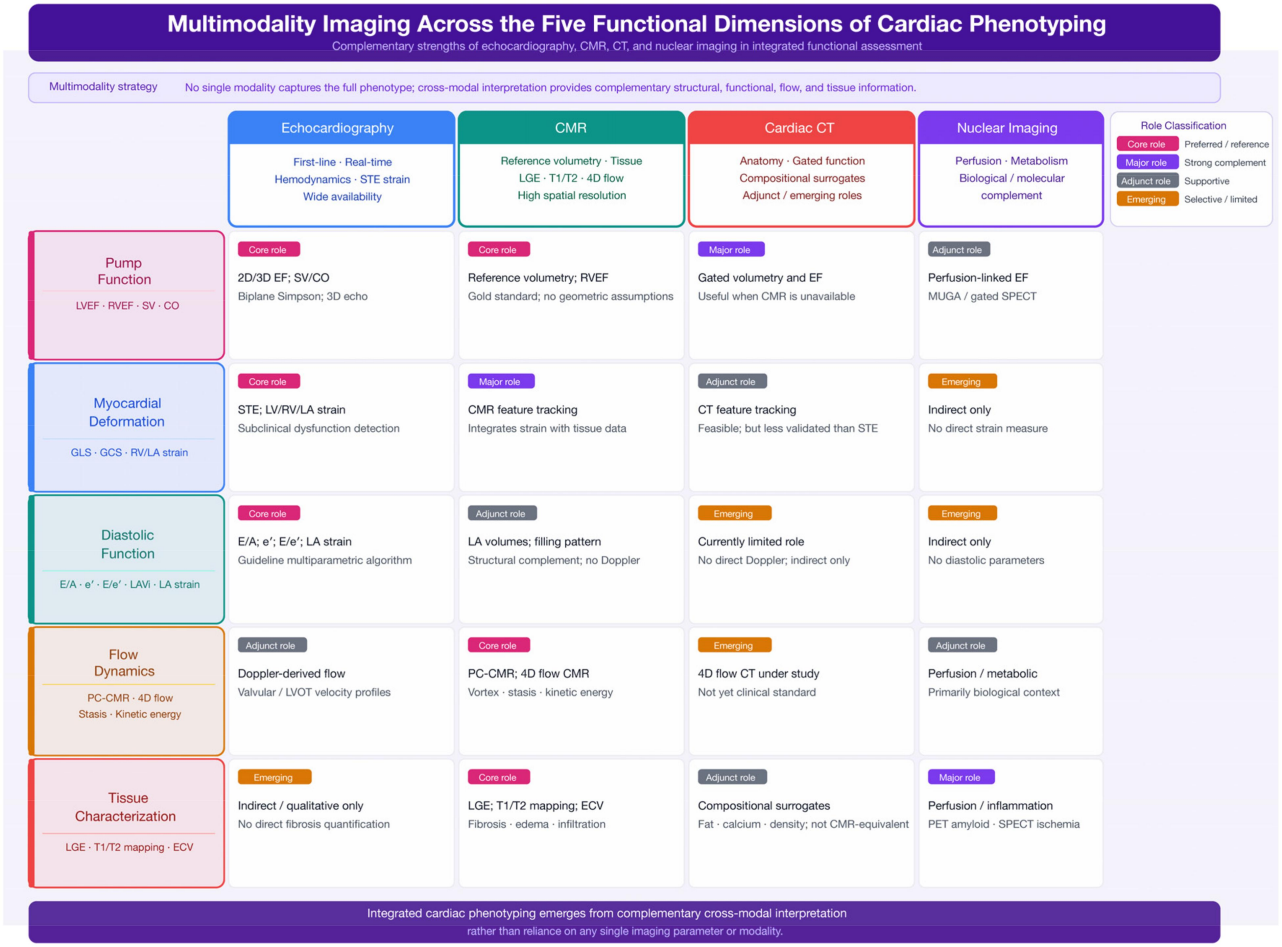
Multimodality imaging across the five functional dimensions of cardiac phenotyping.

Despite their clinical value, these parameters are inherently global and load-dependent, reflecting both contractility and loading conditions rather than intrinsic myocardial mechanics (4, 5, 11). They may therefore remain preserved despite regional dysfunction or early myocardial injury, a limitation most evident in HFpEF and early cardiomyopathy (4, 10, 18). The complementary roles of echocardiography, CMR, CT, and nuclear imaging across the five functional dimensions are summarized in Figure 2.

The complementary roles of echocardiography, cardiac magnetic resonance (CMR), cardiac CT, and nuclear imaging are summarized across five functional dimensions of cardiac assessment: pump function, myocardial deformation, diastolic function, flow dynamics, and tissue characterization. Rather than acting as interchangeable tools, these modalities provide dimension-specific strengths and limitations, with relative contributions categorized as core, major, adjunct, or emerging/selective according to their representative applications within each domain. Different techniques contribute complementary structural, functional, hemodynamic, and tissue-level information, such that integrated cardiac phenotyping depends on cross-modal interpretation rather than reliance on any single imaging parameter or modality.

### 3.2 Myocardial deformation

Myocardial deformation characterizes intrinsic myocardial mechanics by quantifying shortening, thickening, and lengthening along specific axes, detecting abnormalities not reflected by ejection fraction alone (14, 21). Global longitudinal strain (GLS) is the most widely used deformation parameter, while global circumferential strain (GCS) and global radial strain (GRS) provide additional information (14, 21). Because GLS is expressed as a negative value during systolic shortening, its interpretation should focus on GLS magnitude: a less negative value indicates reduced deformation, whereas a more negative value indicates greater deformation. Approximate normal GLS values are commonly around −18% to −22%, although reference ranges vary by vendor, population, loading conditions, and analysis software. Beyond the left ventricle, RV free-wall strain and LA strain are increasingly recognized as markers of chamber-specific dysfunction and prognosis (21–23).

From a modality perspective, speckle-tracking echocardiography (STE) is the established first-line method for routine deformation assessment; CMR feature tracking (CMR-FT) provides complementary high-spatial-resolution analysis with integrated tissue characterization (14, 24); and CT-based feature tracking (CT-FT) remains adjunctive pending further prospective validation (20, 25). The principal strength of deformation imaging lies in its sensitivity for subclinical dysfunction. In the cardio-oncology setting, a relative reduction in GLS magnitude of >15% from baseline, meaning that GLS becomes less negative, serves as an early marker of cancer therapy-related cardiac dysfunction (CTRCD), preceding LVEF decline (5, 21). In cardiomyopathy, strain identifies clinically relevant dysfunction despite preserved systolic function, and feature tracking-derived GLS improves risk stratification beyond conventional models (14, 26, 27). In HFpEF and hypertensive heart disease, impaired LV GLS and LA strain predict adverse outcomes and identify early functional impairment (23, 28, 29). RV and LA deformation further extend the physiological scope of strain imaging, with RV free-wall strain demonstrating incremental value in pulmonary vascular disease and LA strain serving as a sensitive indicator of diastolic burden (6, 22, 23, 30). Vendor-specific algorithm variability and post-processing differences remain the principal technical limitations, particularly for parameters beyond LV GLS (14, 21, 31).

### 3.3 Diastolic function

Diastolic function reflects ventricular relaxation, compliance, and filling under physiological loading conditions, providing direct insight into hemodynamic burden and ventricular–atrial coupling (1, 7, 15). Clinical assessment uses a multiparametric approach integrating mitral inflow velocities (E/A ratio), tissue Doppler-derived annular velocities (e′), the E/e′ ratio, and left atrial volume index (1, 7, 15). Despite guideline-based algorithms, a substantial proportion of patients are classified as indeterminate, and diagnostic accuracy is reduced by confounding factors including age, heart rate, arrhythmias, and valvular disease (15, 32, 33).

LA reservoir strain has emerged as a valuable dynamic marker of atrial compliance and left ventricular filling pressure, improving detection of elevated filling pressures in HFpEF and demonstrating independent prognostic value across cardiovascular conditions (23, 30, 34, 35). In HFpEF, elevated filling pressures and impaired relaxation are primary determinants of exercise intolerance and adverse prognosis despite preserved ejection fraction (2, 10, 18). In terms of modality role, echocardiography remains the primary platform for diastolic assessment, providing the full multiparametric dataset in a single examination; LA strain by speckle-tracking adds incremental diagnostic and prognostic value within the same acquisition; and CMR-derived filling parameters offer complementary information when echocardiographic windows are inadequate or simultaneous tissue characterization is required (12, 13, 15).

### 3.4 Flow dynamics

Flow dynamics extends cardiac functional assessment from myocardial properties to intracardiac blood behavior, directly characterizing blood transport pathways and energy transfer within cardiac chambers (9, 16). Phase-contrast CMR (PC-CMR) enables accurate quantification of forward flow, regurgitant volume, and shunt fraction, and is well established for evaluation of valvular and congenital heart disease (16, 36, 37). 4D flow CMR further allows time-resolved 3D velocity field acquisition, enabling simultaneous assessment of vortex formation, flow stasis, and kinetic energy distribution (16, 38).

Emerging evidence indicates that abnormal flow patterns may be mechanistically linked to disease progression rather than merely reflecting downstream dysfunction. In heart failure, altered vortex organization and increased energy dissipation are associated with impaired ventricular efficiency (38, 39); in valvular heart disease, turbulent kinetic energy and regurgitant jet characteristics may refine severity assessment (36); and identification of flow stasis in the left atrial appendage has been linked to thromboembolic risk (16, 36). From a modality standpoint, PC-CMR is clinically established for quantitative flow measurement, particularly in valvular and congenital heart disease. By contrast, 4D flow CMR provides more comprehensive 3D flow characterization but remains limited in routine practice by longer acquisition times, complex post-processing requirements, inter-vendor variability in acquisition and analysis pipelines, incomplete standardization of reference values, and limited reimbursement pathways in many healthcare systems (16). These constraints currently restrict 4D flow CMR mainly to selected clinical questions, specialized centers, and research-oriented workflows; robust cost-effectiveness data for routine broad implementation remain limited.

### 3.5 Tissue characterization

Tissue characterization provides direct insight into the structural and compositional substrate underlying myocardial performance, enabling identification of fibrosis, edema, inflammation, and infiltration (8, 17, 40). Late gadolinium enhancement (LGE) imaging allows detection and characterization of focal fibrosis and scar; its presence, extent, and pattern are consistently associated with adverse outcomes across cardiomyopathies, ischemic heart disease, and myocarditis (41–44). Parametric mapping techniques—including native T1, T2, and extracellular volume (ECV) quantification—extend this capability to diffuse myocardial alterations with demonstrated diagnostic and prognostic value in cardiomyopathies, myocarditis, cardiac amyloidosis, and metabolic heart disease (8, 17, 45–49).

Tissue abnormalities often precede overt functional impairment: elevated T1 or ECV values may indicate diffuse fibrosis before ejection fraction changes, and elevated T2 reflects active inflammation prior to structural remodeling (46, 50, 51). Integration of tissue characterization with functional parameters enhances phenotyping; combining deformation abnormalities with LGE burden improves risk stratification in cardiomyopathy, while ECV-quantified diffuse fibrosis links tissue pathology to diastolic dysfunction in HFpEF (27, 34, 42, 46). In terms of modality hierarchy, CMR is the central platform for tissue characterization, particularly when myocardial substrate information is expected to clarify diagnosis, refine risk stratification, or guide management, providing LGE, T1, T2, and ECV within a single comprehensive examination; CT contributes selected tissue-related surrogates, including calcium burden, pericardial involvement, delayed iodine enhancement, and CT-derived ECV in specific settings (20); and nuclear imaging—including technetium-based scintigraphy and positron emission tomography (PET)—offers targeted molecular characterization, particularly for cardiac amyloidosis and inflammatory conditions (8, 17).

## 4 Multi-chamber integration: from left ventricular-centered assessment to multidimensional cardiac functional phenotyping

### 4.1 Right ventricular function as a cross-dimensional marker

RV function reflects both intrinsic contractility and pulmonary vascular afterload (52, 53). Conventional indices such as tricuspid annular plane systolic excursion (TAPSE) and RV fractional area change (FAC) are widely used. Compared with these conventional measures, RV free-wall strain provides greater sensitivity for early RV dysfunction and carries independent prognostic value across disease states (6, 22). RV dysfunction is a strong prognostic determinant in pulmonary hypertension, an independent predictor of adverse outcomes in left-sided heart failure, and a key marker of hemodynamic compromise in valvular heart disease (1, 2, 52–55). The right atrium (RA) contributes to RV preload and is incorporated into multi-chamber coupling analyses alongside the LA, LV, and RV.

### 4.2 Left atrial function as an integrator of diastolic burden

The LA modulates ventricular filling across reservoir, conduit, and booster phases, reflecting cumulative diastolic dysfunction (23, 56). In HFpEF, impaired LA reservoir function and reduced LA strain reflect elevated filling pressures and reduced ventricular compliance (23, 30, 35). In atrial fibrillation, impaired LA strain is associated with blood stasis and thromboembolic risk (56), and in hypertensive heart disease, progressive LA remodeling often precedes overt systolic dysfunction (29).

### 4.3 Ventricular-ventricular interaction (LV–RV coupling)

LV–RV coupling, mediated through the interventricular septum, shared myocardial fibers, and pericardial constraint, is particularly relevant in pulmonary hypertension and advanced heart failure, where RV pressure overload and interventricular interaction can impair LV filling and reduce cardiac output (52, 53, 57, 58). In congenital heart disease, dysfunction in one ventricle may secondarily compromise the other through altered loading, remodeling, and septal interaction (59, 60).

### 4.4 Atrial-ventricular coupling (LA–LV interaction)

Atrial–ventricular coupling directly influences ventricular filling and diastolic pressure. The concept of LA–LV mechanical coupling links atrial reservoir, conduit, and booster function to LV relaxation and compliance (61). In HFpEF, impaired LV relaxation and increased stiffness elevate filling pressures and reduce LA compliance (30, 34, 35). In mitral regurgitation, chronic volume overload causes maladaptive LA–LV coupling and progressive dysfunction (55, 62), and impaired LA–LV coupling has been independently associated with reduced exercise capacity and worse clinical outcomes (30, 63).

### 4.5 Integrated perspective of multi-chamber functional phenotyping

**Figure 3:**
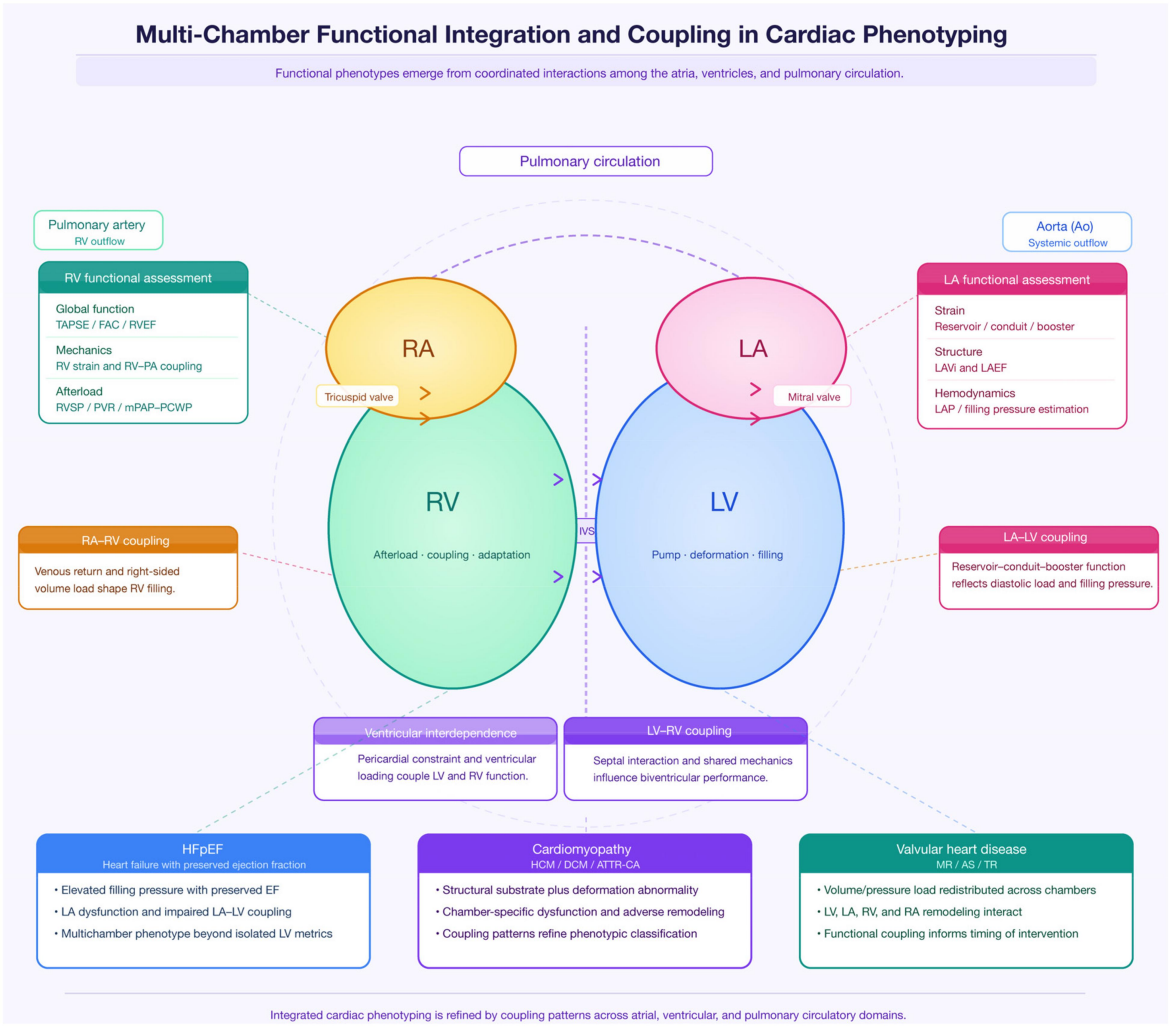
Multi-chamber integration and coupling in multidimensional cardiac functional phenotyping.

Collectively, cardiac function emerges from coordinated interactions across multiple chambers rather than isolated chamber performance (12, 13). In HFpEF, impaired LV relaxation, elevated filling pressures, LA dysfunction, and altered flow dynamics coexist to define the disease phenotype (10, 18, 39). In cardiomyopathies, myocardial fibrosis, deformation abnormalities, and chamber interactions collectively determine progression and prognosis (27, 42). In valvular heart disease, altered loading affects multiple chambers through interdependent mechanisms (55, 62). This multi-chamber perspective is one of the features that distinguishes the proposed framework from conventional single-chamber assessment paradigms and links chamber interaction directly to the escalation strategy outlined in Section 5. The major coupling axes and representative disease-related phenotypes are illustrated in Figure 3.

Coordinated interactions among the right atrium (RA), right ventricle (RV), left atrium (LA), left ventricle (LV), and pulmonary circulation refine multidimensional functional phenotyping beyond isolated chamber-based assessment. Major coupling axes include RA–RV coupling, LA–LV coupling, LV–RV coupling, and ventricular interdependence, each contributing distinct physiological and clinical information. Representative disease-specific phenotypes, including heart failure with preserved ejection fraction (HFpEF), cardiomyopathy, and valvular heart disease, are shown as examples in which chamber interaction patterns provide additional mechanistic and prognostic insight. Double-headed arrows indicate bidirectional mechanical coupling rather than anatomical blood-flow direction, reflecting reciprocal effects among atrial compliance, ventricular relaxation, preload, and afterload.

## 5 Clinical escalation pathway for phenotype-guided multimodality assessment

### 5.1 Multiparametric integration of functional dimensions

Combining parameters across different functional domains demonstrates incremental clinical value. Integration of LVEF, GLS, LA function, and ECV enables simultaneous assessment of pump function, myocardial mechanics, diastolic burden, and structural substrate; studies have consistently shown that combining deformation and tissue parameters improves diagnostic accuracy and risk stratification compared with either alone (27, 42, 46). Similarly, combining diastolic indices (E/e′), LA strain, and flow-related parameters improves estimation of filling pressure and hemodynamic burden in complex scenarios such as HFpEF (34, 35, 39). The purpose is not to collect more parameters, but to determine whether the available abnormalities point to a coherent functional phenotype with clinical consequences.

### 5.2 Functional phenotyping in specific disease states

The concept of functional phenotyping is especially relevant in heterogeneous syndromes such as HFpEF, where no single parameter defines disease severity. HFpEF is characterized by a constellation of abnormalities—impaired myocardial deformation, elevated filling pressures, LA dysfunction, diffuse fibrosis, and altered flow dynamics (10, 39)—and integrating these parameters identifies distinct phenotypes with different clinical outcomes (18, 64). In cardiomyopathies, integration of deformation imaging, tissue characterization, and chamber interaction parameters improves risk stratification (12, 27, 42), while in valvular heart disease, combining flow quantification with ventricular function and tissue markers optimizes assessment of disease severity and intervention timing (55, 65). Across these disease states, the added value of multimodality assessment is greatest when standard echocardiography leaves clinically relevant uncertainty regarding myocardial substrate, chamber interaction, flow burden, or prognosis.

### 5.3 Stepwise escalation from echocardiography to advanced imaging

Clinical multimodality integration should be synergistic rather than merely additive. As outlined in the Introduction, standard echocardiography serves as the first-line gatekeeper for cardiac functional assessment. Advanced echocardiographic analysis, including GLS, RV free-wall strain, LA strain, and 3D volumetric assessment, should be added when conventional echocardiographic indices are discordant with symptoms, when early myocardial dysfunction is suspected despite preserved LVEF, or when chamber-specific dysfunction may influence prognosis or treatment decisions.

Escalation to CMR is most appropriate when myocardial substrate information or highly reproducible volumetric assessment is expected to change clinical interpretation. Examples include suspected cardiomyopathy, myocarditis, infiltrative disease, unexplained HFpEF, complex RV morphology, poor echocardiographic acoustic windows, or discordance between symptoms and conventional echocardiographic findings. In these settings, CMR can integrate ventricular volumes, feature-tracking strain, LGE, T1/T2 mapping, and ECV to distinguish functional impairment from structural myocardial disease.

**Figure 4:**
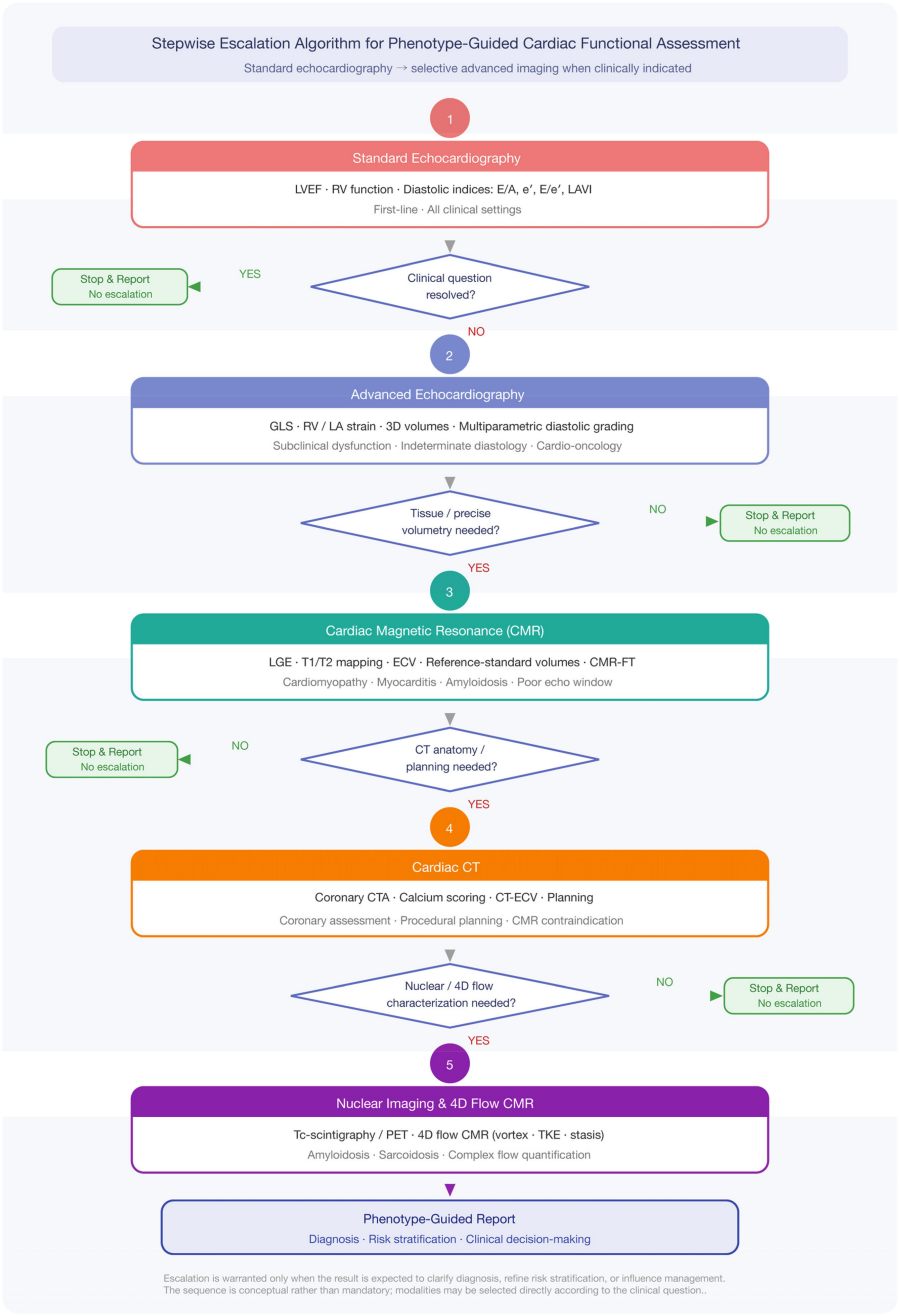
Stepwise escalation algorithm for phenotype-guided cardiac functional assessment.

CT should be used selectively when anatomical definition, coronary assessment, valvular or vascular calcification, procedural planning, or CMR contraindication is clinically relevant. Nuclear imaging and PET provide targeted molecular or metabolic information, particularly in suspected cardiac amyloidosis, inflammatory cardiomyopathy, or viability assessment. PC-CMR is established for quantitative flow assessment in valvular and congenital heart disease, whereas 4D flow CMR should be reserved for selected scenarios in which conventional flow quantification is insufficient or discordant, such as complex valvular regurgitation, congenital heart disease, abnormal great-vessel flow, or research-oriented assessment of intracardiac flow energetics. A stepwise clinical decision algorithm integrating these escalation principles is presented in Figure 4.

The algorithm illustrates a selective escalation pathway from standard echocardiography to advanced echocardiography, CMR, cardiac CT, nuclear imaging, and 4D flow CMR according to unresolved clinical questions. Standard echocardiography serves as the first-line gatekeeper, whereas advanced imaging is added only when the expected information may clarify diagnosis, refine risk stratification, guide procedural planning, or influence management. The sequence is conceptual rather than mandatory; specific modalities may be selected directly according to the clinical question, local expertise, availability, and patient-specific constraints. CMR, cardiac magnetic resonance; CTA, computed tomography angiography; ECV, extracellular volume; GLS, global longitudinal strain; LAVI, left atrial volume index; LGE, late gadolinium enhancement; PET, positron emission tomography; TKE, turbulent kinetic energy.

### 5.4 Disease–dimension matrix and clinical interpretability

**Figure 5:**
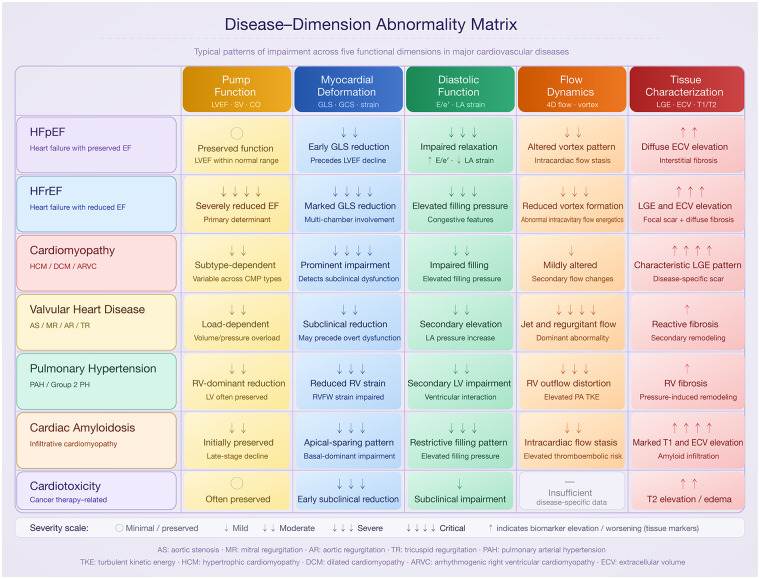
Disease-dimension abnormality matrix in multidimensional cardiac phenotyping.

Viewed across diseases, this integrated approach suggests that major cardiovascular diseases can be reinterpreted as distinct multidimensional phenotypes rather than as entities defined by single metrics or isolated chamber abnormalities. The disease–dimension abnormality patterns arising from this framework are summarized in Figure 5. This matrix should be interpreted as a conceptual synthesis, not as a validated scoring system or a set of universal quantitative diagnostic thresholds. To improve clinical interpretability while preserving the conceptual nature of the matrix, representative guideline- or consensus-based reference values for selected biomarkers are summarized in Table 1. These values should be considered approximate reference anchors rather than disease-universal cut-offs, because thresholds may vary according to disease context, imaging modality, vendor platform, acquisition protocol, loading conditions, and patient characteristics. Data-driven analysis methods that enable the fusion of high-dimensional imaging biomarkers into coherent phenotypic models are discussed in the following section.

For each of seven representative cardiovascular diseases—HFpEF, heart failure with reduced ejection fraction (HFrEF), cardiomyopathy, valvular heart disease, pulmonary hypertension, cardiac amyloidosis, and cardiotoxicity—the matrix displays the relative prominence of abnormality across pump function, myocardial deformation, diastolic function, flow dynamics, and tissue characterization, using a semiquantitative severity scale; upward arrows denote biomarker elevation or worsening for tissue-based parameters. This matrix is intended as a conceptual synthesis of representative disease patterns rather than a validated scoring tool, and the displayed severity levels should not be interpreted as universal diagnostic thresholds. Approximate reference anchors for selected biomarkers are provided in Table 1 to improve clinical interpretability; these values should be applied in the context of disease setting, imaging modality, vendor platform, acquisition protocol, loading conditions, and patient characteristics. The matrix supports a phenotype-oriented interpretation of cardiac dysfunction organized by multidimensional abnormality patterns rather than isolated chamber metrics.

## 6 Artificial intelligence-enabled multimodality functional phenotyping

The rapid expansion of quantitative imaging has markedly increased the dimensionality of cardiac functional data. In this context, artificial intelligence (AI)-based methods may help operationalize multimodality phenotyping by standardizing measurements, integrating heterogeneous imaging features, and identifying reproducible disease subgroups (66–68).

### 6.1 Automated quantification and reproducibility

Deep learning algorithms have demonstrated high accuracy in automated segmentation of cardiac chambers across echocardiography, CMR, and CT, enabling rapid extraction of volumetric and functional parameters including LVEF, ventricular volumes, and myocardial strain (20, 69–71). Automated analysis substantially reduces interobserver variability—a recognized limitation of conventional measurements, particularly for deformation parameters such as GLS and RV strain (31, 69, 72)—supporting more standardized and reproducible cardiac assessment in routine clinical practice.

### 6.2 Phenotype classification and outcome prediction

Beyond measurement, data-driven approaches enable identification of distinct functional phenotypes. In HFpEF, phenomapping—a data-driven clustering approach applied to multiparametric imaging data—has identified patient clusters characterized by varying degrees of myocardial fibrosis, diastolic dysfunction, and ventricular–atrial interaction, each associated with different clinical outcomes (18, 64). Machine-learning analysis of 3D RV motion has also enabled outcome prediction in pulmonary hypertension, illustrating how motion signatures can extend beyond conventional chamber metrics (73). Models combining GLS, LGE/ECV, LA function, and clinical variables have demonstrated improved performance over conventional risk scores in heart failure and cardiomyopathy (27, 64, 66, 74), and AI-based approaches integrating flow, ventricular function, and structural parameters have shown promise in predicting disease progression in valvular heart disease (65, 75).

AI-based multimodal integration may be particularly valuable in diagnostically challenging infiltrative and inflammatory cardiomyopathies, where subtle and overlapping abnormalities across functional, tissue, and molecular domains can be difficult to synthesize using conventional sequential assessment. In cardiac amyloidosis, AI-assisted integration of CMR-derived ECV, native T1 mapping, longitudinal strain, wall-thickness patterns, and nuclear scintigraphy findings may help combine structural, functional, and molecular information into a more coherent diagnostic and risk-stratification profile (46, 47, 66). In cardiac sarcoidosis, where LGE pattern, T2 signal, metabolic activity on PET, and RV involvement require joint interpretation, AI-based fusion approaches may help prioritize patients for further evaluation and support arrhythmic-risk assessment, although prospective validation remains limited (44, 68). These disease contexts exemplify scenarios in which the burden of multiparametric interpretation is high and the potential clinical return of AI-assisted integration may be greatest. External validation and standardization remain essential before widespread clinical implementation (66, 67).

### 6.3 AI as an operational tool for multimodality integration

AI-based methods may support the construction of comprehensive functional phenotypes by combining parameters across myocardial deformation, diastolic function, flow dynamics, and tissue characterization (66–68). In practice, such tools might first alert clinicians to unexpected discordance between echocardiographic and CMR strain values, or identify patients in whom additional tissue characterization is most likely to alter therapeutic strategy.

Realizing this potential requires explicit attention to the interpretability of AI-derived outputs. Current deep learning models used in cardiac imaging frequently operate as black boxes: their predictions may be accurate at the population level but provide limited mechanistic rationale accessible to clinicians, creating accountability gaps in cases of diagnostic error and barriers to clinical governance, regulatory review, and institutional adoption of AI-based medical devices (66, 67). Explainability methods—including attention-based visualization, saliency maps, and SHAP (SHapley Additive exPlanations) value analysis—can partially address this limitation by identifying which imaging features most influence model output, but their clinical interpretability and robustness across imaging platforms remain inconsistent (67, 68). Cross-platform generalizability poses an additional challenge: models trained on data from a single vendor or institution may underperform when applied to external datasets, and prospective multicenter validation remains limited for most published AI cardiac imaging tools (66, 67, 76). Until interpretability, generalizability, and regulatory standards are more fully established, AI-derived phenotypic outputs should be regarded as decision-support adjuncts rather than autonomous diagnostic conclusions.

## 7 Challenges and translational pathways

Despite rapid parameter expansion, current research remains largely focused on individual metrics rather than unified cross-dimensional frameworks (12, 13, 39). Functional domains have developed in parallel, producing a growing inventory of measurable parameters without a corresponding structure for their coordinated interpretation. The primary challenge is therefore not further parameter development, but the construction of coherent integrative models applicable consistently across imaging platforms and clinical settings.

Measurement standardization represents a closely related barrier. Metrics such as GLS, ECV, and LA strain lack harmonized acquisition protocols and reference values across vendors and centers, limiting their transferability in multicenter environments (14, 21, 46). Substantial inter-vendor variability affects not only deformation parameters but also emerging techniques such as 4D flow CMR, where differences in velocity encoding, post-processing algorithms, and reference datasets limit cross-center comparability (16). Even where reproducibility is established within a single modality, the direct clinical meaning of multidimensional parameter combinations—how they should influence diagnosis, risk stratification, or treatment selection—remains inadequately defined. Advancing the clinical interpretability of multiparametric outputs, including the transparency of AI model-derived scores, is a prerequisite for broader adoption in routine imaging practice (66, 67).

Resource constraints and cost-effectiveness represent a distinct but equally important translational barrier. Advanced multimodality imaging—particularly CMR with parametric mapping, 4D flow CMR, and nuclear imaging—involves acquisition time, specialized technical expertise, post-processing capacity, and infrastructure requirements that are not uniformly available across healthcare settings. In high-volume centers and resource-constrained environments, the incremental diagnostic and prognostic value of each additional modality must be weighed against its cost, time requirements, and competing demands on imaging resources (76, 77). Reimbursement frameworks for advanced techniques such as 4D flow CMR and AI-based phenotyping tools remain absent or inconsistent across many healthcare systems, creating a practical implementation barrier even when technical feasibility has been demonstrated. A clinically credible multidimensional framework must therefore not prescribe indiscriminate multimodality use, but instead provide explicit guidance on when the incremental value of advanced imaging justifies its resource cost—a principle embedded in the stepwise escalation strategy proposed in this review.

Workflow integration presents an equally important obstacle to clinical translation. Most available evidence originates from single-center or retrospective studies with limited external generalizability (69, 76). More critically, multiparametric approaches have not been embedded in established clinical pathways; guideline-driven decision-making in heart failure and valvular disease continues to rely on conventional volumetric metrics, while structured imaging interpretation, standardized acquisition protocols, and interdisciplinary communication pathways for multidimensional assessment remain absent from most clinical environments (1, 55). Embedding this framework into routine imaging workflows will require prospective validation in multicenter cohorts that reflect the diversity of real-world practice, the development of standardized acquisition and post-processing pipelines across modalities, and the integration of multidimensional outputs into clinical workflows that deliver interpretable, clinician-facing phenotypes rather than unstructured parameter lists (76, 77).

Progress on these fronts will require coordinated efforts across imaging vendors, clinical trialists, health economists, and regulatory bodies to move multidimensional phenotyping from academic demonstration to routine clinical implementation.

## 8 Conclusion

Cardiac functional assessment is undergoing a paradigm shift from single-parameter evaluation toward multidimensional cardiac functional phenotyping. Although LVEF remains clinically relevant, it is insufficient to capture the complexity of cardiac physiology. Advances in multimodality imaging—including echocardiography, CMR, CT, and nuclear techniques—now enable characterization across five interconnected domains: pump function, myocardial deformation, diastolic function, flow dynamics, and tissue characterization, with further refinement through multi-chamber integration.

The framework proposed in this review differs from conventional multimodality practice by organizing imaging assessment around functional phenotypes rather than modality-specific parameters. It also embeds a stepwise escalation strategy in which advanced imaging is added only when specific clinical questions—clarifying diagnosis, refining risk stratification, or influencing management—remain unresolved after first-line assessment. This approach is intended to support clinically actionable interpretation while remaining sensitive to cost, workflow constraints, and the practical realities of diverse healthcare environments.

AI-based methods may help translate this framework into reproducible measurement, parameter fusion, and phenotype discovery, but their clinical value will depend on model interpretability, cross-platform generalizability, and prospective validation. Moving beyond isolated metrics toward structured, phenotype-guided functional assessment may improve disease characterization and clinical decision-making across cardiovascular care—provided that implementation is governed by clinical need rather than imaging availability.
